# Short and long term impact of adenotonsillectomy on the immune system

**DOI:** 10.5935/1808-8694.20130006

**Published:** 2015-10-14

**Authors:** Fábio Pires Santos, Raimar Weber, Bibiana Callegaro Fortes, Shirley Shizue Nagata Pignatari

**Affiliations:** Former Fellow in the Otology and Ear Surgery service at UNIFESP/EPM, Specialist on ENT by the Brazilian ENT-HNS Associaton (ENT in the ENT Clinic of the Professor Edmundo Vasconcelos Hospital); PhD in ENT by FMUSP, former Fellow of Facial Plastc Surgery at FMUSP, Specialist on ENT by the Brazilian ENT-HNS Associaton (ENT in the ENT Clinic of the Professor Edmundo Vasconcelos Hospital); First-year Resident Physician in the ENT Clinic of the Professor Edmundo Vasconcelos Hospital; PhD in Medicina by UNIFESP/EPM, MSc in ENT by UNIFESP/EPM, Specialist on ENT by the Brazilian ENT-HNS Associaton, Adjunct Professor in the Department of ENT at UNIFESP/ EPM (ENT in the ENT Clinic of the Professor Edmundo Vasconcelos Hospital). ENT Clinic of the Professor Edmundo Vasconcelos Hospital

**Keywords:** adenoidectomy, child, immune system

## Abstract

Palatine and pharyngeal tonsils are immune reactive lymphoid organs that manifest specific antibodies and B/T-cell activity to respond to a variety of antigens. They perform humoral and cellular immune functions. The possible effects of adenotonsillectomy upon the immune system remain controversial.

**Objective:**

To study the short and long-term impacts of tonsillectomy upon the cellular and humoral immunity of children.

**Method:**

This longitudinal prospective study included 29 children referred to adenotonsillectomy for adenotonsillar hypertrophy. Serum IgA, IgM, and IgG and lymphocyte counts were analyzed at three points in time: before surgery, 1-2 months after surgery (short term), and 12-14 months after surgery (long term).

**Results:**

TCD4+ cell counts were significantly increased shortly after surgery. IgA and IgG values were significantly reduced in the long run, but were within normal ranges for this age group.

**Conclusion:**

This study indicated that adenotonsillectomy does not pose negative short or long term impacts upon the cellular and humoral immunity of children submitted to the procedure.

## INTRODUCTION

The palatine and pharyngeal tonsils, along with the lingual and tubal tonsils and the lateral pharyngeal bands are the Waldeyer's ring most important structures^1^^,^^2^. They are secondary lymphoid organs part of the mucosa-associated lymphoid tissue (MALT), which present immune activity mainly between four and ten years of age^2^.

Strategically located at the common entry point of the respiratory and digestive systems, they are the first point of contact between our bodies and a wide range of food and airborne antigens and microorganisms^3^, ^4^, ^5^, ^6^.

From the histological point of view, the tonsils are organized in a manner closely related to their function in the immune system, and are divided into four compartments, namely the reticular crypt epithelium, the extrafollicular area, the mantle zones of lymphoid follicles, and the follicular germinal centers^1^, ^2^, ^3^.

The reticular crypt epithelium is made up by a system of 10 to 30 tonsil crypts that go from the surface to deeper levels^1^, ^2^, ^3^. Additionally, it hosts a complex system of specialized cells - M-cells and antigen-presenting cells (APC) - designed to imprison antigens and transport them through the epithelial barrier^1^, ^2^, ^3^^,^^7^.

In the extrafollicular area, dendritic cells and macrophages process antigens and present them to T-cells, which differentiate mainly into cytokine-producing T helper cells (CD4+), and cytotoxic or effector T-cells (CD8+)^1^, ^2^, ^3^^,^^7^.

In the follicular germinal centers, B-cells stimulated by T helper cells (CD4+) and cytokines start proliferating and turning into plasmocytes, which results in the production of all sorts of immunoglobulins (IgG: ∼65%, IgA: ∼20%, IgM, IgD, IgE), expansion of memory B-cell clones or migration of B-cells to distal sites. The overflow of blood lymphocytes onto the palatine tonsils and vice-versa is essential for the immune competence of these organs. The mantle zones are usually close to the crypt epithelium and contain predominantly naive B-cells, which are probably responsible for local immunity^1^, ^2^, ^3^^,^^7^.

Knowledge gathered on Waldeyer's ring physiology and evidences on the contributions it makes to immune local and systemic response have been the topic of controversy, in particular due to the injudicious manner in which adenotonsillectomy procedures have been carried out along the years. Thus, further research is required to analyze the possible immune adverse impact consequent to the procedure.

More recently, a wide range of studies have shown that the resolution of upper airway obstructions in pediatric patients after adenotonsillectomy has led to improved sleep and PSG test results, phonation with clearer and better voice, more significant growth and weight gain, resolution of nocturnal enuresis and behavior and neurocognitive disorders, and improved quality of life^8^.

Although adenotonsillectomy is the most commonly performed procedure in children^7^^,^^9^^,^^10^, its true impact upon the pediatric immune system is yet controversial. Few studies have looked into this matter to consider what happens to patients in the long run.

Therefore, this study aimed to verify the short and long-term impact of adenotonsillectomy by analyzing serum markers for cellular and humoral immunity in children.

## METHOD

This longitudinal prospective study was approved by the institution's Research Ethics Committee and given permit 0582/07.

The parents or guardians of the subjects were informed of the purposes of this study, the tests patients would be offered, and the possible risks inherent to participating in the study. Patients were only included in the study after their parents or guardians had read and signed an Informed Consent Term.

### The patients

This study was the continuation of another study started back in 2008. Therefore, it reflected the long-term follow-up findings of some patients enrolled in the initial stages of a research project.

The initial stage of the study started in the period between May and October of 2008, as 29 children aged between two and eight years (16 males and 13 females; mean age: 4.5 years) were enrolled. The patients were followed up by physicians from the ENT clinic. Subjects had been diagnosed with palatine and pharyngeal tonsil hypertrophy and prescribed adenotonsillectomy.

Patients were initially followed up for one to two months after surgery. At the end of this period they were assessed for short-term immune adverse impacts.

Still in this stage, the 29 subjects were divided into two groups based on their ages:

• Group I - children under four years of age.
• Group II - children aged four and above.

In the second stage of the study patients were tested again 12 to 14 months after surgery. Fourteen of the 29 patients in the original group were tested again to verify the occurrence of adverse immune impact. This time they were not divided into different groups.

Personal and family history of immune deficiency or diseases with known immune-related etiology served as exclusion criteria.

All patients were submitted to adenotonsillectomy with general anesthesia and orotracheal intubation. Tonsils were removed through cold dissection and adenoids using Beckman curettes.

### Lab workup

Lab tests included humoral immunity serum marker levels - IgA, IgM, and IgG - and absolute T helper cell (TCD4+) and cytotoxic T-cell (TCD8+) counts.

Samples were collected at three different times: before surgery during anesthesia induction (sample 1); one to two months after surgery (sample 2); and 12 to 14 months after surgery (sample 3).

The two first samples were collected for the 29 individuals enrolled in the first stage of the study. ANOVA tests were carried out on the data sets collected before surgery vis-a-vis short-term follow-up.

Paired data for the three samples (before surgery, short-term follow-up, and long-term follow-up) were obtained for 14 of the 29 patients originally enrolled.

Immunoglobulin serum levels were determined through turbidimetry (Wiener^®^ - Argentina). Subpopulations of lymphocytes were identified by flow cytometry (Beckman Coulter^®^ - U.S.). Immunoglobulin serum levels and absolute lymphocyte counts varying up to two standard deviations above or below the age-specific mean values were considered normal. Mean values reported in the literature were considered as reference values, as provided by the Pathology Lab at the Edmundo Vasconcelos Hospital, where all samples were tested.

### Statistical analysis

Software package SPSS^®^ version 16.0 (Chicago, U.S.) was used in the statistical analysis of the data. Variations on TCD4+ and TCD8+ counts and serum levels of IgA, IgM, and IgG before surgery and short-term follow-up (samples 1 and 2/n = 29) were compared using Student's *t*-test for paired samples. The Kolmogorov-Smirnov test was used to check the sample's normal distribution.

The Friedman test was used to compare the three samples (n = 14).

Differences were statistically significant when *p*-values were under 0.05 (5% significance level).

## RESULTS

Serum levels of cellular (absolute TCD4+ and TCD8+ counts) and humoral (IgA, IgM, IgG serum levels) immunity parameters and the mean variations between samples 1 & 2 of the 29 patients can be seen on Table 1. In the initial stage of the study, in which 29 patients were enrolled, considering the global variation between samples 1 & 2, there was a statistically significant increase of 186 cells/mm^3^ in the absolute TCD4+ count (*p* < 0.05). TCD8+ counts were slightly reduced in sample 2, yet within normal ranges. Serum levels of IgA, IgM, and IgG in sample 2 were not significantly increased (Table 1).

**Table 1:** Cellular and humoral immunity parameters, before surgery and during short-term follow-up (one to two months after surgery) (n = 29).

	Before surgery	Short-term follow-up	Difference (95% Cl)	*p*
LTCD4+	1.140 ± 563	1326 ± 507	186 (24-348)	0.026^a^
LTCD8+	693 ± 294	675 ± 294	–18 (-119-82)	0.715
IgA	120 ± 59	126 ± 62	6 (-1-15)	0.094
IgM	122 ± 61	127 ± 57	5 (-2-12)	0.169
IgG	1004 ± 209	1010 ± 235	6 (-49-59)	0.840

Data presented as mean ± standard deviation.

a *p* < 0.05 (*Student's t*-test for paired samples): comparison between values before and after surgery.

When patients were analyzed separately based on their ages, subjects on group I (under four years of age) had lower TCD8+ counts and IgG serum levels, albeit not significantly. TCD4+, IgA and IgM were increased, but not significantly.

Patients on group II (four years and above) had increased values in all assessed parameters, but statistical difference was seen only for TCD4+ count, with a mean increase of 230 cells/mm^3^ (*p* < 0.05) (Table 2).

**Table 2:** Cellular and humoral immunity parameters for group II, before and after surgery. (n = 15).

	Before surgery	After surgery	Difference (95% Cl)	*p*
LTCD4+	981 ± 369	1211 ± 509	230 (27-433)	0.029^a^
LTCD8+	663 ± 236	720 ± 335	57 (82-195)	0.394
IgA	160 ± 42	170 ± 40	10 (5-24)	0.178
IgM	131 ± 80	135 ± 74	4 (7-15)	0.435
IgG	1127 ± 161	1149 ± 189	22 (44-88)	0.485

Data presented as mean ± standard deviation.

a *p* < 0.05 (*Student's t*-test for paired samples): comparison between values before and after surgery.

The analysis of the results obtained for the group with 14 patients, for whom three samples were collected, indicated a statistically significant reduction on IgA and IgG levels in the long-term follow-up samples against preoperative and short-term follow-up samples. However, these values were still within normal range. TCD4+ and TCD8+ counts were slightly increased in the long-term follow-up samples, but failed to achieve statistical significance (Table 3).

**Table 3:** Cellular and humoral immunity parameters before surgery, during short-term follow-up (1 to 2 months) and long-term follow-up (12 to 14 months). (n = 14).

	Before surgery	Short-term follow-up	Long-term follow-up	*p*
LTCD4+	1165 ± 382	1374 ± 555	1545 ± 542	0.110
LTCD8+	647 ± 393	630 ± 367	786 ± 225	0.210
IgA	136 ± 54	137 ± 53	106 ± 43	0.026^a^
IgM	140 ± 83	145 ± 73	120 ± 69	0.110
IgG	1070 ± 179	1037 ± 190	918 ± 182	0.002^a^

Data presented as mean ± standard deviation.

a *p* < 0.05 (Friedman test): comparison between values before surgery and values observed in short and long-term follow-up.

None of the long-term follow-up patients had altered immunoglobulin or lymphocyte levels in their long-term samples except for one subject, whose IgA levels were slightly below reference values before surgery and were unaltered 14 months into follow-up.

## DISCUSSION

The adverse immune impact of adenotonsillectomy in children has been studied for some time. Key studies connected to this topic were analyzed (Table 4).

**Table 4:** Effects of adenotonsillectomy upon humoral and cellular immunity. Literature review.

Reference/year	N	Controls	Age (years)	Follow-up (w, m, y)	Postop humoral immunity	Postop cellular immunity
Gogoi D^14^, 1979	10	20	5-14	3 m	No significant alteration	ND
Lal H^11^, 1984	40	40	5-15	1 m	^−^IgG, to normal levels	ND
Cantani^13^ A, 1986	65	65	2-11	1-4 m	^−^IgA, IgM & IgG	ND
Bussi M^15^, 1991	40		3-30	3 m	ND	CD3+, CD4+, CD8+ & B-cells
Friday G^12^, 1992	152	116	18-16	7-30 m	^−^IgG	ND
Böck A^16^, 1994	160	302	4-8	6 m-11 y	^−^IgA	CD21+ & CD4+
Rio-Navarro B^20^, 1995	33		3-13	1 w - > 12 w	^−^IgA (1-4 w);^−^IgG (> 12 w), to normal values	ND
Mira JGS^17^, 1998	30	30	3-15	1 & 12 m	No significant alteration	ND
Redondo F^15^, 2000	117			1-6 m	No significant alteration	ND
Jurkiewicz B^3^, 2002	80	40	3-14	1 & 6 m	^−^IgA, IgM, IgG (1 m); IgA, IgM, IgG (6 m), to normal values	^−^CD8+ (1 & 6 w), to normal values
Ikinciogullari A^4^, 2002	15	15	4-10	1-1.5 m	No significant alteration	^−^CD19+; CD3+, CD8+CD25+, CD19+CD23+
Kaygusuz I^9^, 2003	37	35	5-9	1 m	^−^C3, C4, IgA, IgM, IgG; to normal values	CD4+; −CD25+
Sebastiá L^21^, 2004	89		4-10	1 & 4 m	^−^IgA, IgG (1 & 4 m); to normal values	ND
Akker E^18^, 2006	63	60	2-8	12 m	^−^IgA, to normal values	ND
Faramarzi A^10^, 2006	102		2-15	2 & 8 w	IgA (2 w); became normal (8 w)	CD17+ (2 w); (8 w)
Baradaranfar M^6^, 2007	30	30	4-10	6 m	^−^IgG, to normal values	TCD3+, TCD8+, BCD20+
Kaygusuz^19^, 2009	20	20	10-14	54 m	No significant alteration	^−^CD4 & CD19; CD16+65+ & CD25+

Postop: postoperative; w: weeks; m: months; y: years; ND: not defined.

Diverging reports have been presented in regards to short and long-term postoperative changes in immunoglobulin levels.

Veltri et al.^22^ reported statistically significant decreases - though within normal range - in IgG levels, whereas IgA, IgM, and IgD remained unaltered. Lal et al.^11^ also found reduced IgG levels, although not lower than controls (*p* < 0.01). The same outcomes were reported by Friday et al.^12^ in the medium-term follow-up of adenotonsillectomy patients when compared to controls monitored clinically. The incidence of upper airway infections was not increased among patients in this study.

Cantani et al.^13^ and Kaygusuz et al.^9^ noticed a significant medium-term follow-up decrease in IgA, IgM and IgG and concluded that the removal of the tonsils was not merely an anatomy-related event. Zielnik-Jurkiewicz et al.^3^ documented transient reductions in IgA, IgM and IgG (one month) followed by recovery onto normal values (six months). El-Ashmawy et al.^23^ followed patients for two months and saw significant reductions in IgA and IgG levels.

Unlike others, Gogoi et al.^14^, Redondo et al.^24^ and Ikinciogullari et al.^4^ did not find significant changes in immunoglobulin serum levels after adenotonsillectomy.

On the other hand, the effects upon cellular immunity have not been extensively studied and few publications on the topic are available^4^.

Baradaranfar et al.^6^ and Bussi et al.^15^ described significant increases in postoperative activated T and B cell counts. Ikinciogullari et al.^4^ observed reduced CD19+, and increased CD3+, CD8+CD25+, CD19+CD23+ counts.

In a paper published in 2003, Kaygusuz et al.^9^ found significant increases in TCD4+ and reduced CD25+ counts one month after surgery.

Decreased TCD8+ counts were reported by Zielnik-Jurkiewicz et al.^3^ at one and six months into follow-up, albeit in levels comparable to controls.

The long-term effects of adenotonsillectomy upon immunoglobulin levels and lymphocyte counts have been less studied, and few are the studies available on the topic.

Friday et al.^12^ did not report long-term alterations (16 to 30 months) in IgA, IgM, and IgG levels in patients aged between one and 16 years. In a study with 106 patients followed up for a mean 6.6 years, Böck et al.^16^ published slight increases in CD21+ counts and reduced levels of CD4+ and IgA, all of which statistically significant. However, increases in the incidence of upper airway infections were not reported.

In 1996, Mira et al.^17^ followed 30 Brazilian patients aged between three and 15 years for one to 12 months. Minor reductions were reported on immunoglobulin levels, but they were not statistically significant.

Akker et al.^18^ analyzed 123 adenotonsillectomy patients for 12 months and reported significant decreased in IgA levels, although still within normal range.

In a study published in 2009, with the first part of the follow-up period published in 2003^9^, Kaygusuz et al.^19^ reported, after 54 months of follow-up, statistically significant increases in CD4+ and CD19+ and reductions in CD16+56+ and CD25+ counts. No long-term alterations were found in immunoglobulin levels.

Antibody production by the tonsils was first observed in 1958 and, since then, they have been considered as active immune lymphoid organs, which manifest specific antibodies and T and B-cell activity in response to a wide range of antigens, performing humoral and cellular immunity functions^3^^,^^16^.

Diseases related to the tonsils are among the most common reasons why people see ENTs, with respiratory obstruction atop the list of complaints^7^.

Delayed diagnosis and treatment of these diseases may result in important consequences such as behavior alterations, low growth and weight gain, craniofacial alterations secondary to mouth breathing, mastication and swallowing disorders, in addition to cor pulmonale and left heart failure^8^.

Nonetheless, within the last few years the literature has described the benefits of adenotonsillectomy for children with sleep-related obstructive respiratory disorder^8^.

Despite the many papers published within the last three decades on the immune impacts of adenotonsillectomy in pediatric patients, there is no definite evidence that the procedure impairs the immune system.

However, still today patient family members and even physicians tend to believe that the removal of palatine and pharyngeal tonsils may result in impaired immunity.

A great deal of the controversy surrounding possible local or systemic immune involvement is derived from the comparison between the removal of chronically infected and/or hypertrophied tissue and the possible consequences of the elimination of an important defense barrier from the host^4^.

In order to facilitate the understanding of our results, the study was divided into two stages: the initial stage, in which short-term immune impacts of adenotonsillectomy (one to two months into follow-up) were analyzed for all children originally enrolled in the study; and the complementary stage, in which immune impacts 12 to 14 months after surgery were analyzed using the same lab tests in 14 of the 29 original subjects.

Considering the analysis of the data from the first stage, no statistically significant differences were seen in IgA, IgM, and IgG levels.

Short-term follow-up TCD4+ counts were increased by a mean 186 cells/mm^3^ in relation to preoperative values (*p* < 0.05).

**Graph 1:**
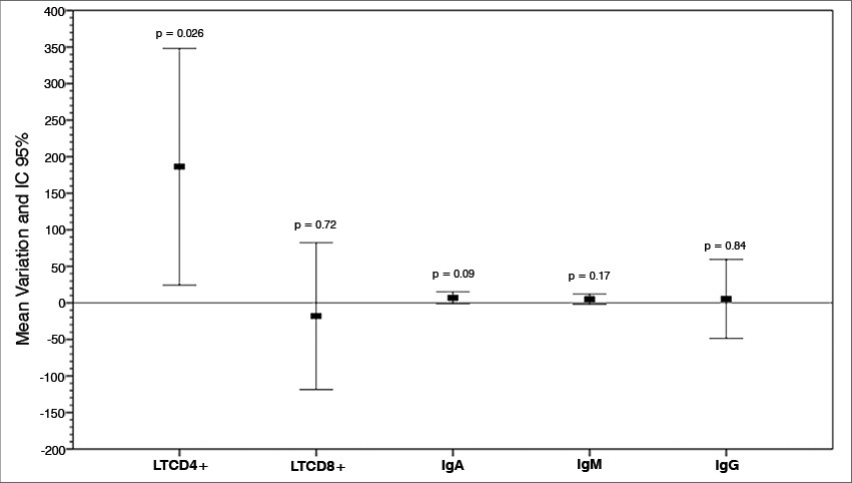
Humoral and cellular immunity serum markers. Mean postoperative variation and 95% CI. LTCD4+: Lymphocytes TCD4+; LTCD8+: Lymphocytes T CD8+; IgA: Immunoglobulin A; IgM: Immunoglobulin M; IgG: Immunoglubulin G.

Graph 1 shows the mean variation between the parameters acquired before surgery and during short-term follow-up and their respective *p* values.

In order to investigate the possible impact of adenotonsillectomy upon the immune system of patients in various age ranges, the 29 subjects were further divided into two groups: one for individuals under four years of age (n = 14), and another with subjects aged four and above (n = 15).

No relevant immune impairment was seen in either of the groups separately. On the contrary, there was a significant increase in TCD4+ counts in Group II subjects (Table 2), who would theoretically be at a higher risk, as in this age range tonsil immune activity is more prominent.

**Graph 2:**
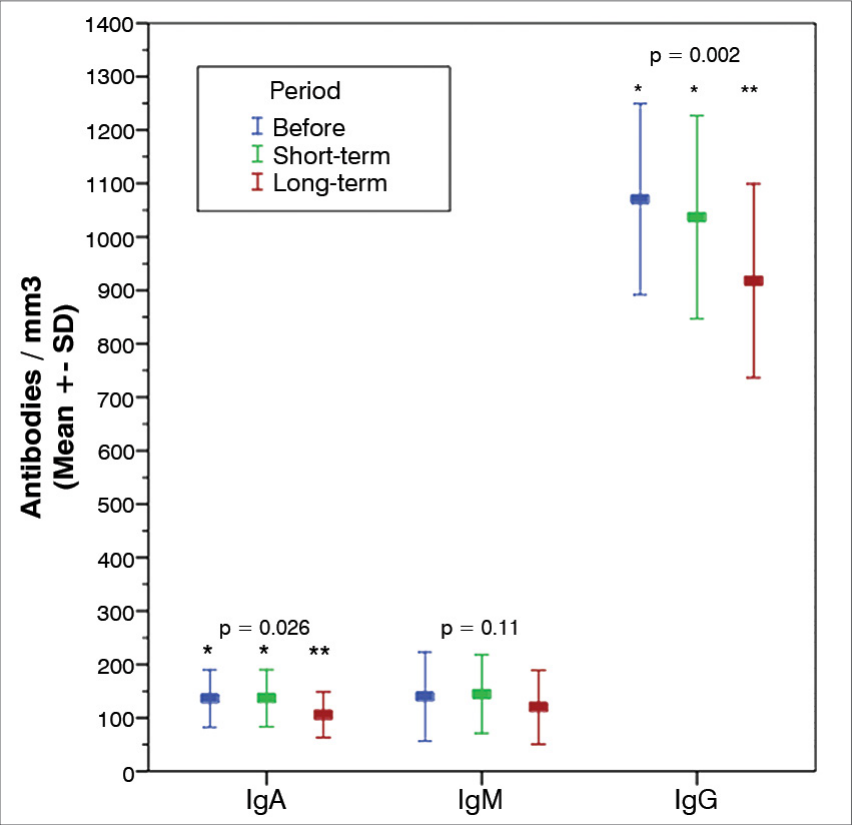
Humoral immunity serum markers. Mean variation in the recent (1 to 2 months) and late (12 to 14 months) postoperative periods and 95% CI. IgA: Immunoglobuline A; IgM: Immunoglobuline M; IgG: Immunoglobuline G.

The analysis of long-term follow-up samples of the 14 patients enrolled in the complementary stage of the study revealed a statistically significant reduction on IgA and IgG levels when compared to preoperative and short-term follow-up levels (Graph 2 and Table 3). Differently from short-term follow-up values, no significant alterations in TCD4+ and TCD8+ counts were seen (Table 3).

Our results agree with the literature, despite the significant divergence seen in the observations reported by other authors.

In general, more recent publications reported transient reduced antibody production (IgA and IgG in particular), though within normal range. Only two publications^16^^,^^19^ looked into the long-term variations in lymphocyte count and none showed adverse immune impact.

The origin of these alterations is still uncertain, but they may be related to surgical stress, reduction of immunoglobulin-producing lymphoid tissue, and reduction of antigen load^3^^,^^6^^,^^9^^,^^12^^,^^18^^,^^21^. Additionally, these alterations have not adversely impacted the frequency of occurrence or the severity of upper airway infection, differently from what was previously believed^12^^,^^18^^,^^25^.

Akker et al.^18^, on a controlled study (adenotonsillectomy *vs.* clinical treatment), suggested that the remaining lymphoid tissue may compensate for the removal of the tonsils, once differences were not found between the values seen for both groups.

Nonetheless, it is believed that when these organs are involved with chronic inflammation, they do not perform a relevant role in protecting against upper airway infection, but inhibit immune response and increase infection severity^3^^,^^7^^,^^9^.

TCD4+ and TCD8+ are involved in the regulation of B-cell response, which by its turn results in the production of immunoglobulins. In the extrafollicular zone, where antigens are processed, approximately two thirds of the T-cells are TCD4+. In situations of infection, however, marked proliferation of cytotoxic T-cells (TCD8+) has been observed with a consequent reversal on the TCD4+/TCD8+ ratio, resulting in early local and late systemic suppression of antibody production. B-cell activation is affected, thus leading to lesser availability of these cells. TCD4+ B-cell dependent response could also be impaired^3^^,^^6^.

Other important findings in the literature indicate increased immunoglobulin and lymphocyte levels before surgery, supposedly due to chronic antigen stimulation of the tonsils^3^^,^^9^^,^^11^^,^^22^^,^^25^. Some authors consider it an important marker for disease, to assist in the decision of offering adenotonsillectomy to the patient at hand^3^^,^^9^.

From the global standpoint, therefore, current evidence points to insignificant alterations in lab test results and failure to show that the removal of the tonsils leads to compromised immunity. Thus, still today the immune function of the pharyngeal and palatine tonsils and the possible effects of adenotonsillectomy upon the immune system are a matter of controversy.

Our complementary results reinforce the early findings of our study, which indicate that adenotonsillectomy does not significantly impair immune function. Serum marker levels, albeit reduced, were kept within normal range. This is highly relevant in respect to the realm of clinical practice, as this finding becomes an important tool in advising patient family members and informing other assisting physicians over the actual impact of surgery.

Lastly, it is worthwhile making a few comments on the outcomes of this study. The difficulty keeping in touch with the patients in the long run prevented the enrollment of all 29 children in the complementary stage of the study. The reasons for this shortcoming include the H1N1 pandemics that manifested more intensely at the time the third sample was collected, thus driving people away from hospital settings, and the marked improvement from symptoms patients experienced after surgery. We opted to keep the data on the patients who did not take part in the last stage of the study to perform short-term analysis of variation and produce more meaningful results. The smaller sample in the end of the study did not allow subjects to be divided into different age groups.

The inclusion of a control group could have improved the legitimacy of our results.

## CONCLUSION

The adenotonsillectomy pediatric patients enrolled in this study did not present short or long-term adverse impacts upon their cellular or humoral immunity. Therefore, the procedure did not result in patient immune deficiency.
